# Design, implementation and evaluation of e-learning program for common diseases to smartphone-based medical students: at a developing university

**DOI:** 10.1186/s12909-023-05023-4

**Published:** 2024-01-10

**Authors:** Elham niromand, Meysam Siyah Mansoory, Ghobad Ramezani, Mohammad Rasool Khazaei

**Affiliations:** 1 Fertility and Infertility Research Center, Health Technology Institute, Kermanshah University of Medical Sciences, Kermanshah, 0000-0001-6406-6779 Iran; 2 Department of Biomedical Engineering, School of Medicine, Kermanshah University of Medical Sciences, Kermanshah, 0000-0002-8160-7618 Iran; 3 Education Development Center, Kermanshah University of Medical Sciences, Kermanshah, 0000-0002-8192-5587 Iran; 4 Fertility and Infertility Research Center, Health Technology Institute, Kermanshah University of Medical Sciences, Kermanshah, 000000021965099x Iran

**Keywords:** E-learning, Common diseases, Innovative teaching methods, Mobile-based education, Online learning

## Abstract

**Objective:**

Mobile-based educational software offers a wealth of resources that can foster the growth of learners and facilitate the creation of an interactive learning environment. This environment encourages both students and instructors to engage in exploration and the examination of various medical issues. The objective of this study is to design, implement, and evaluate an electronic educational program focused on common medical conditions, specifically tailored for medical students and accessible through mobile phones.

**Method:**

The study was conducted following an action research approach, which comprised four key stages: needs assessment, application design, training, and evaluation. This research took place at the Kermanshah University of Medical Sciences’ Medical School. In the needs assessment phase, a formal survey was distributed to the teaching faculty members, requesting them to identify diseases and medical issues of high importance for medical interns’ education that were suitable for virtual teaching. Each faculty member was asked to prioritize a minimum of three and a maximum of seven cases. Subsequently, 10 faculty members from various departments completed the survey, leading to the identification of 47 common diseases after eliminating duplicates. These 47 cases were then presented to 30 medical interns, who were asked to select the 20 most significant cases. The 20 diseases with the highest statistical frequency were selected for further development due to resource constraints. The mobile application was developed for the Android platform using the Java programming language and the Android Studio development environment. To assess the application’s effectiveness from the students’ perspective, a questionnaire was designed, encompassing 25 questions across five domains: satisfaction, performance, learning, usability, and educational effectiveness. The questionnaire employed a Likert scale, with response options ranging from ‘completely disagree’ to ‘completely agree,’ scored from 1 to 5. One hundred medical interns and trainees were invited to participate in the evaluation, with 92 of them completing the questionnaires.

**Results:**

The findings revealed a significant disparity in the average scores between students who underwent traditional teaching methods and those who engaged in mobile-based app-assisted education. This discrepancy was statistically significant across all three examined components.

**Conclusion:**

Mobile-based learning represents a burgeoning educational approach with profound implications for healthcare education and the enhancement of patient care quality. The widespread integration of mobile phones into the educational framework offers a flexible teaching paradigm, fostering the potential for continuous lifelong learning.

## Introduction

On March 11, 2020, the World Health Organization (WHO) declared COVID-19 a global pandemic. In response to the rapid global spread of the disease, governments worldwide implemented unprecedented measures related to social distancing in an effort to control the spread of the virus. These measures included the temporary closure of in-person classes, leading to a transition of educational institutions to virtual teaching [1]. Mobile Learning (M-Learning) entails the delivery of educational content through portable devices such as laptops, smartphones, and handheld devices, allowing learners to engage with their studies at any point in the learning process. Extensive research has indicated that electronic education offers numerous advantages, including enhanced learning quality, convenient access to a vast reservoir of information, rapid information retrieval, cost-efficiency, improved content accuracy, academic advancement for both students and educators, interactive teaching methods that encourage collaboration, and the effective utilization of appropriate educational technologies [2]. This approach aligns well with contemporary education, providing both learners and instructors with enhanced flexibility and accessibility [3]. What medical students learn today will directly impact their ability to meet the healthcare needs of patients in the not-so-distant future. Thus, the quantity, quality, and nature of the education provided to medical students undeniably play a pivotal role in furthering the goals of the healthcare system. Presently, electronic systems for delivering medical science education, encompassing fields like medicine, nursing, and pharmacy, are widely adopted. The expansion and evolution of electronic technologies in the realm of medical science education span various domains and are experiencing rapid, global growth [4].

Today, smart phones with faster processors, higher memory and smaller batteries have made significant changes in a harmonious relationship with efficient operating systems that are capable of performing advanced functions. Every day, the role of mobile phones in the educational curriculum becomes more prominent. Due to the impact of the comprehensive use of smart phones on the communication between health professionals and evidence-based medicine, the use of these phones by people working in the field of health can lead to the improvement of medical care standards [5]. Aligned with healthcare policies, the realization of the WHO’s objectives, and the pursuit of national healthcare system policy-making initiatives, there is an imperative to prioritize the enhancement of general practitioners’ abilities as the primary providers of patient care. This necessitates improvements in their education during academic studies and continuous virtual training. Within the nation, the adoption of electronic content in medical student education has encountered limited enthusiasm among both professors and students, primarily due to software and hardware complexities. Meanwhile, foreign examples face substantial challenges marked by high-dollar costs and severe limitations. It is noteworthy that, despite recent endeavors by medical universities to incorporate electronic education, it has primarily taken the form of creating class content through voice-over slides in the past few years. Despite having the requisite knowledge and technological infrastructure within the nation, the effective implementation of multimedia educational content for students remains largely untapped. Therefore, the decision was made to utilize the mobile application designed for teaching 15 common diseases to medical students. Furthermore, the transition towards virtual education and electronic learning is an inevitable progression. The COVID-19 pandemic has presented an opportunity to take significant steps in enhancing student education through the use of new technologies, enabling us to discern the strengths and weaknesses inherent in this educational approach. Therefore, the purpose of this study was to design, implement and evaluate an e-learning program for common diseases for medical students based on smartphones. Considering that 8 diseases were known as common diseases, in this study, we sought to design and implement electronic education for these diseases using smartphones to make the education effective and improve its efficiency.

## Methods

### Study design

This study was conducted using an action research approach, divided into four phases: needs assessment, application design, training, and evaluation. The research was conducted within the premises of the Kermanshah University of Medical Sciences’ Medical School. Action research is an approach in qualitative research that helps teachers and educational agents to somehow integrate research with the educational situation so that they can play a direct and immediate role in the educational situation and improve the education process. reach the desired point. Also, McNiff (1991) mentions the purpose of action research by teachers to study and solve classroom problems by using scientific methods [6].

### Study setting

#### Phase 1: needs assessment

##### A- identifying the Learners’ needs

In order to identify the educational needs of learners, a structured form was designed and distributed to faculty members who expressed interest in education, representing various teaching departments. These faculty members were requested to prioritize a list of diseases and related medical issues, including signs and symptoms, deemed significant for the education of medical interns and suitable for virtual teaching. Each faculty member was required to list a minimum of three and a maximum of seven items. Ten faculty members from diverse teaching departments completed the provided form, resulting in the identification of 47 common diseases after eliminating duplicate entries. Subsequently, these 47 diseases were presented to 30 medical students (interns), who were tasked with selecting the 20 diseases they considered most vital. Based on the frequency of responses, the 20 diseases with the highest statistical prevalence were determined. Finally, taking into account financial and time constraints, the researchers selected 15 common diseases for content production through the application.

##### B- determining Educational needs

The determination of educational needs was a critical phase, recognizing that the creation of electronic education demands expertise from a variety of specialists, including instructors, instructional designers, media developers, and editors. This stage aimed to structure the educational program and define its content. It also involved the assessment of tools for content creation, encompassing graphic design, animations, sound, and images.

#### Phase 2: application design

##### A- Content Development

Building upon the needs assessment, which identified 15 common diseases based on the input of learners and faculty members, the development of educational content commenced. This phase included the filming and editing processes.

##### B- validation of Course Content

The recorded content underwent a validation process where it was reviewed by the relevant instructors before final production. In a collaborative effort within the team, they meticulously examined the content from both scientific and other pertinent perspectives. This review process resulted in necessary revisions and enhancements. Some instances required re-recording, while others involved meticulous editing, with all feedback and suggestions from the instructors thoughtfully incorporated into the final content.

**Fig. 1:**
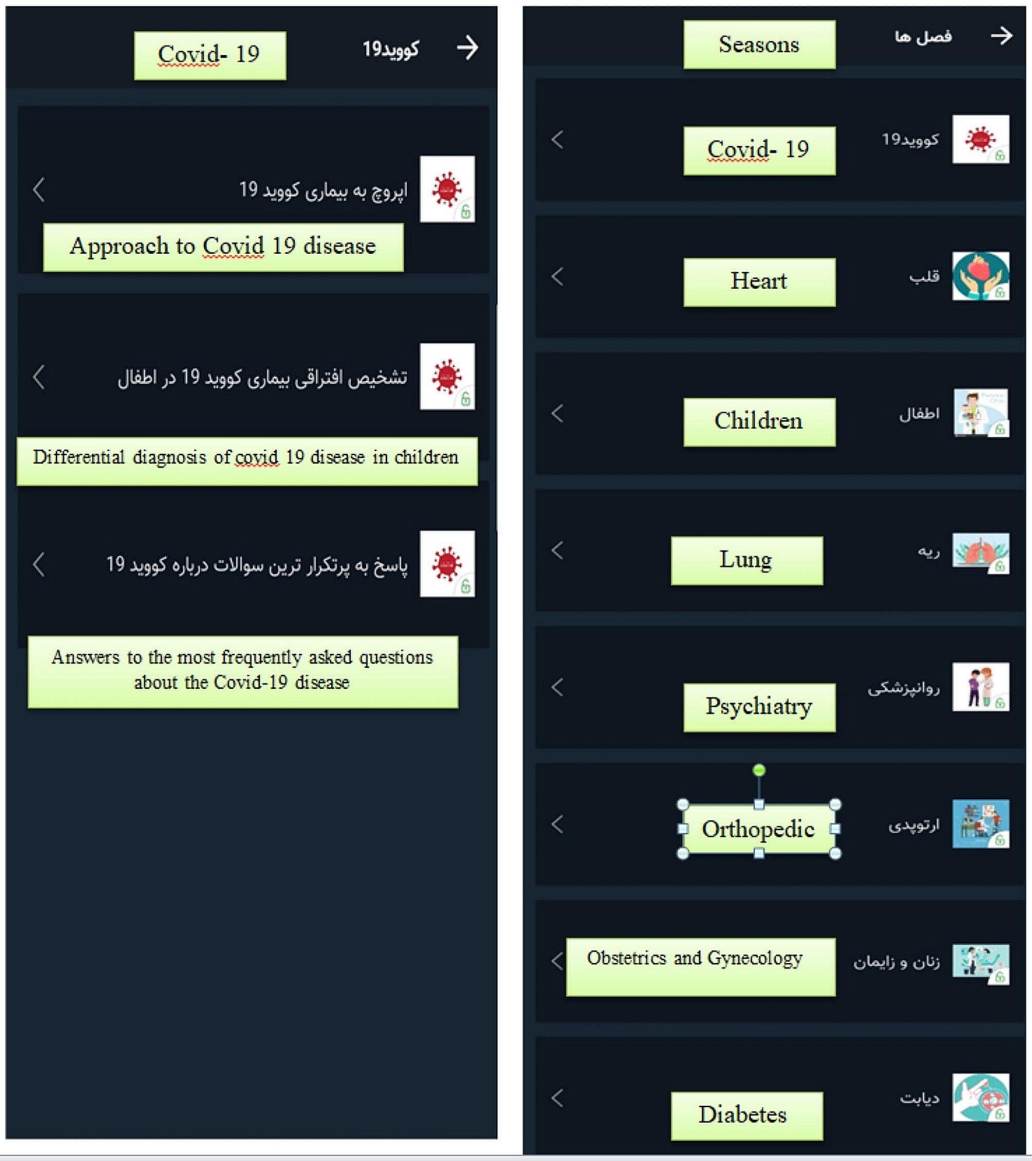
Categories of Lessons

*C- Application Design*: The application design was carried out in three steps: (1) First Step (Server): All elements, including the logic, scenarios, textual content, images, videos, and more, that users encounter within the application were stored on the server. This content was subsequently delivered to the mobile app through programming. On the server, the technology employed was ASP.NET MVC, implemented with C# and HTML. The database utilized was MS SQL, and communication between the server and the application was facilitated through two-way encryption. (2) Second Step (Application): To facilitate the interaction with the system (comprising the server, application, and management panel), a client software, in this case, a mobile app, served as the interface between the user and the system. This Android application was developed using the Java programming language within the Android Studio development environment. The application was designed to fulfill two primary objectives: education and examination. Education section of the application was organized into eight general chapters, encompassing topics like COVID, Cardiology, Pediatrics, Pulmonology, Psychiatry, Orthopedics, Obstetrics & Gynecology, and Diabetes. Each topic featured educational content, including text and video materials. Following completing each educational topic, users were presented with a multiple-choice exam. The system immediately scored the exam, and users received a comprehensive report. Furthermore, users could access their historical results and monitor their progress within a specific topic by visiting the “Report” Sect. 3) Third Step (Management Panel): The system administrator possessed the capability to upload new content or make modifications to existing content through the management panel. This panel was constructed on the web platform using ASP.NET MVC technology and was divided into four sections: Categories of Lessons, Educational Topics, Exams, and User Management.(Attachment N.1), (Fig. 1).

#### Phase 3: training

##### A- video production and approval

Following extensive efforts, including coordination with instructors, the filming team, physical space preparation for filming, multiple recording sessions, and meticulous content editing to ensure quality, the educational video clips were produced. Once they received approval from the Deputy of General Medicine at the Faculty, they were made accessible for student education.

##### B- creation of a virtual Learning Group

A virtual Telegram group was established for students, and the educational content was shared within this group.

##### C- Mobile Application Development

Furthermore, in collaboration with faculty members from the Biomedical Engineering department and the software development team, a mobile application was meticulously designed for installation on mobile phones. This application incorporated educational content, including the design of quizzes and exams as integral features within the app.

### Data collection

In the fourth phase of the study, following conducting the educational course, the students’ learning levels were assessed using the mobile application. A total of 50 internship students were divided into two groups: the first group comprised 25 students who received traditional teaching, while the second group, of the same size, received education through the mobile application. To ensure standardization, the examination questions were derived from previous years’ pre-internship exam questions. In this manner, 60 questions were selected and categorized into three topics: pediatric diseases, cardiology, and respiratory diseases, with each topic containing 20 questions. Each question carried a score of one, allowing students to achieve a score between zero and twenty. The acquired scores from the exam were then input into SPSS-16 software. The mean scores of the students were compared using parametric tests, considering the normal distribution of the data. The results revealed that the average scores of students who received traditional teaching were lower than those who received education through the mobile application. This discrepancy in averages was statistically significant across all three topics, as indicated in (Table 1).

#### Results (phase 4)

**Table 1:** Comparison of Mean Scores between Two Groups of Internship Medical Students with Conventional and Mobile-Based App (Med ED) Education

	Conventional Education	Mobile-Based Education	P
	Mean ± Standard Deviation	Mean ± Standard Deviation	
Children	14.1 ± 64.78	15.1 ± 92.50	0.008
Cardiovascular	13.2 ± 66.25	15.1 ± 12.61	0.012
Respiratory and Breathing	13.2 ± 96.09	15.1 ± 36.58	0.01

The results obtained based on the study objectives are as follows:

Achievement of the First Objective (Needs Assessment): As delineated in the activities section, the first objective of the study involved conducting a comprehensive needs assessment among the learners to discern their educational requirements. This process resulted in the identification of fifteen common diseases for content creation, along with the establishment of the instructional program’s structure.

Results for the Second and Third Objectives (Design and Evaluation): Subsequently, in line with the second and third study objectives, the specific instructional content was systematically created and subjected to validation through the feedback of subject matter experts in their respective fields. Following this, the content was meticulously designed by specialists to facilitate the development of an Android application. As a part of the evaluation process, a questionnaire was distributed to students, and their feedback was collected across five key areas: satisfaction, performance, learning, usability, and academic-instructional quality. The comprehensive results underscored the students’ positive perception of the designed application, as presented in Table 1.

Results for Achieving the Fourth Objective: As previously detailed, education was imparted to the students, and a comprehensive survey and examination were administered as part of the assessment process.”

Results for Achieving the Fifth Objective: In this phase, the level of learning among students who received instruction through the designed application was assessed through a written examination. Their scores were subsequently compared with those of students who received traditional instruction. The findings revealed a notable increase in the scores of students in the group that received education via the mobile application, in comparison to those who received traditional instruction. The complete results can be found in Table 2.

For assessing the application from the students’ perspective, a comprehensive questionnaire was meticulously crafted, encompassing 25 questions distributed across five key domains: satisfaction, efficiency, learning, usability, and scientific-educational. The Likert scale was employed, allowing respondents to rate the questions on a scale ranging from ‘completely disagree’ (1) to ‘completely agree’ (5). The questionnaires were disseminated among a total of 100 interns and trainees for evaluating the application, and 92 students diligently completed and returned them. Within the trainee student group, the highest scores were achieved in the following order: satisfaction, usability, learning, efficiency, and educational quality, with an overall score surpassing 4. In a similar vein, among the trainee students, the highest scores were observed in the order of satisfaction, usability, learning, scientific-educational, and efficiency, with an overall score exceeding 4. The results were consistent among intern students, except for the scientific-educational domain, where the scores were notably higher (Table 2).

**Table 2:** Evaluation of the Application by Interns and Trainees Students

Evaluation Areas	Training course	Internship course
	Mean ± Standard Deviation	Mean ± Standard Deviation
Satisfaction	4.48 ± 0.32	4.48 ± 0.3
Efficiency	3.98 ± 0.44	3.98 ± 0.44
Learning	4.08 ± 0.43	4.1 ± 0.43
Usability	4.12 ± 0.05	4.11 ± 0.5
Scientific - Educational	3.98 ± 0.62	4.01 ± 0.46
Overall Score	4.1 ± 0.32	4.13 ± 0.28

## Discussion

The purpose of the present study was Design, implementation and evaluation of e-learning program for common diseases to smartphone-based medical students. The findings revealed a significant disparity in the average scores between students who underwent traditional teaching methods and those who engaged in mobile-based app-assisted education. This discrepancy was statistically significant across all three examined components. Therefore, the presented data is a new contribution to the current research because it emphasizes how much the educational program affects students.

Lao et al. conducted a randomized controlled trial with a single-blind approach in Spain. Their study demonstrated the efficacy of a mobile phone application as a complementary tool for traditional education, aimed at enhancing ultrasound imaging and tactile skills among physiotherapy students. A total of 49 students participated in this study, and the findings strongly supported the effectiveness of the software used as an adjunct to traditional teaching methods in improving shoulder ultrasound and tactile skills in physiotherapy students [7].

Numerous factors influence the effectiveness of teaching and the attainment of learning objectives, and one pivotal aspect is the choice of teaching methods adopted by instructors. Within educational programs, two overarching teaching models emerge: the instructor-centered model, where the instructor takes a central role in molding behavior according to predetermined patterns, often leading to rapid forgetting of material by learners. In contrast, the student-centered model places a strong emphasis on considering the learners’ needs and capabilities. Within the realm of medical education, there is an increasingly recognized need for a shift in teaching and instructional methods. This transformation has garnered attention in universities worldwide. Both in Iran and across the globe, various studies are underway to explore the impact of diverse teaching methods on the educational landscape.

Sohrabi et al. (2023) concluded in a study that the design and implementation of patient management application and body language had a significant effect on the experimental group [8]. Nerissa Naidoo et al., concluded In total, %70 students responded to the survey assessing perception toward DL (Kirkpatrick’s Level: 1). results showed that the DL-framework was positively received by students and attested that students had an enriched learning experience, which promoted collaborative-learning and student-autonomy. For, Kirkpatrick’s Level: 2 i.e., cognitive development, we compared the summative assessment performance in the H&N course across three cohort of students. The results show that the scores of the cohort, which experienced the course entirely through DL modality was statistically higher (*P* < 0.01) than both the other cohorts, indicating that shift to DL did not have an adverse effect on students’ learning [9]. Moulaei et al(2021) concluded Out of the 66 information needs that were identified via the questionnaire, 58 were considered in designing the application. Features of the designed application were placed in 5 categories: User’s profile, lifestyle, disease prevention and control, application capabilities and user’s satisfaction. The capabilities of the application consist of introducing specialized COVID-19 medical centers, search for the location of medical centers and doctors’ offices, drug management, drug allergies, self-assessment, stress reduction and control, nutrition and diet management, sleep management, doctor’s appointment reminders, communication with other patients and physicians, application settings. Pregnant women rated the usability of the application at a good level. The designed application can reduce the anxiety and stress due to preeclampsia feel and also improve their knowledge as well as attitude towards the COVID-19 pandemic and preeclampsia [10].

The findings study Muhammad Thesa Ghozali(2022) confirmed that the educational app consisted of education, a symptom checker, a list of vaccine information links, the latest news, and COVID-19 statistics. The validity assessment showed that the educational app in this study was very appropriate to be utilized as a digital medium for patient education. In addition, it was also confirmed that all the functions of the app worked well, and participants strongly agreed that the educational materials and features of the app were interesting and helped them to learn COVID-19 prevention easily. It could be concluded that the app could be used as a learning medium for patient education. Further studies, however, were needed to prove its effectiveness in the real clinical world [11]. Zare et al. (2023), in study concluded The designed app was given to 20 people including nutritionists and parents with children under 18 years of age for conducting usability evaluation. According to the scores of participants about the usability evaluation of the app, it can be concluded that groups participating in the study could use the program, and they rated the app at a “good” level. Overall performance of the app, screen capabilities, terms and information of the program, learnability, and general features are scored higher than 7.5 out of 9 [12]. In study Singh et al., Findings show that smartphones and related medical education apps are widely used by medical students and improve their educational experiences. Universities should develop a policy regarding smartphone usage for academic purposes [13].

In the study conducted by Shanmugapriya et al. (2023), nursing students demonstrated a positive disposition toward the utilization of smartphones [14]. The results of Asadullah et al. (2023) strongly suggest that the developed application is both acceptable and usable for nursing instructors and students. Consequently, it serves as an effective tool for advancing and enhancing nursing education and quality within clinical settings [15]. The results of the study conducted by Soyoung Jang et al. (2022) offer promising insights into the use of a competency assessment system in nursing. This system leverages mobile technology and multimedia to evaluate the performance of students in nursing exercises. The study’s findings indicate that a multimedia competency assessment system, when compared to text-based mobile assessments, offers a more realistic, interactive, and satisfying experience. As a result, it is anticipated that such systems will find a place in future nursing curricula to augment students’ nursing competencies [16]. The results of a systematic study suggest that online higher education programs play a pivotal role in advancing mobile interactive learning. They facilitate content creation, communication, and collaboration among learners and instructors, thereby significantly enhancing learning effectiveness. Additionally, despite the well-established use of this technology in higher education, its educational potential for actively engaged instructors remains an appealing prospect [17]. In another study, the findings indicate that the utilization of podcasts has the potential to not only complement instructional methods but also serve as a valuable tool in clinical nursing education. Furthermore, given the aging population in both Eastern and Western countries, podcasts may emerge as an effective means of health education, particularly for older individuals with declining vision and those with visual impairments [18]. The results of a study conducted in Norway offer valuable insights into the creation of an evidence-based technological tool designed to assist nursing students, nursing educators, and clinical preceptors. This program shows promise in addressing multiple challenges reported in clinical nursing education. Following a meticulous development process, the initial prototype of the TOPP-N guidance and assessment app is now poised for testing in subsequent intervention studies [19]. The findings of the study conducted by Sherrel Smith et al. (2023) elucidate the substantial impact of using this program on compassion satisfaction, compassion fatigue, and mindfulness within a limited sample. This study marks the first attempt to evaluate the influence of a mobile phone meditation program on these concepts among acute care nurses. The conclusions drawn from this research point to avenues for further exploration and practical application, such as conducting larger and more diverse sample studies and comparing various applications of meditation [20]. Considering the versatile nature of mobile phone-based learning and its capacity to enhance and enrich learning across all academic disciplines, it occupies a distinct place in education. The widespread integration of mobile phones in the learning process not only augments flexibility in learning but also fosters the culture of lifelong learning. Smartphone learning fosters discussion, communication and collaboration, and assures better student engagement. The millennial learners prefer student-centered, active, and self-directed learning. Smartphone learning has shifted the location of education settings outside of classrooms, demanding a suitable shift in delivery methods. Mobile learning can be integrated into medical education to assess its effectiveness; learning materials should be in a digital format as far as possible to fuel smartphone learning and online education in smart campuses [21–23].

### Limitation & recommendations

One of the most important limitations of this study is the lack of financial support, because these projects are costly and can cover a large area if accompanied by sufficient financial support. In the future, it is suggested that interventional studies be conducted to investigate and compare traditional education and electronic education through various applications in the field of common diseases. It is also suggested that this study be conducted on a larger number of medical students.

## Conclusion

This study has explored the efficacy of mobile-based learning in the field of medical sciences, demonstrating its positive influence on student performance across various facets of medical education. Notable enhancements have been observed in areas such as learning outcomes, practical skills, academic achievements, and the flexibility of study locations. It is evident that mobile-based learning constitutes a pivotal element in medical education, necessitating continual and sustainable enhancements, particularly to meet the educational needs of medical students who require a mode of instruction that is both flexible and accessible. The results further indicate that students who engaged with mobile-based education through an application consistently achieved significantly higher average scores in comparison to their peers who underwent traditional forms of instruction. This substantial disparity in average scores persisted across all three components assessed, unequivocally affirming the effectiveness of mobile-based learning in the realm of medical education.

## Data Availability

The datasets used and/or analyzed during the current are available from the corresponding author on reasonable request.

## Declarations

The present work is the outcome of a research proposal from Kermanshah University of Medical Sciences.
